# Metabolic obesity phenotypes and risk of ischemic stroke: The Rural Chinese Cohort Study

**DOI:** 10.3389/fnut.2025.1749472

**Published:** 2026-01-12

**Authors:** Lu Cao, Lin Zhu, Wei Zhang, Ming Zhang, Dongsheng Hu, Que Wang

**Affiliations:** 1 Department of Anesthesiology, Pain and Perioperative Medicine, The First Affiliated Hospital of Zhengzhou University, Zhengzhou, Henan, China; 2 Department of Biostatistics and Epidemiology, School of Public Health, Shenzhen University Health Science Center, Shenzhen, Guangdong, China

**Keywords:** cardiovascular risk factors, cohort study, ischemic stroke, metabolic obesity phenotypes, nutrition epidemiology

## Abstract

**Background:**

The association of metabolic obesity phenotypes and ischemic stroke remains controversial. We aimed to determine the risk of ischemic stroke according to metabolically healthy and obesity status.

**Methods:**

A total of 14,707 study participants from The Rural Chinese Cohort Study (RCCS) who were free of stroke and cardiovascular disease (CVD) were stratified by metabolic risk and body mass index (BMI) status at baseline during 2007–2008 and further followed up during 2013–2014. Hazard ratio (HR) and 95% confidence intervals (CIs) of incident ischemic stroke associated with different metabolic-obesity categories were estimated by using Cox regression analysis.

**Results:**

During a median follow-up of 6.02 years, we identified 522 newly diagnosed ischemic stroke cases. Risk of ischemic stroke was increased with MHO (HR: 1.87; 95%CI: 0.25–14.18), metabolically unhealthy normal weight (MUNW; HR: 2.36, 95%CI: 1.40–3.98), and metabolically unhealthy obesity (MUO; HR: 3.22, 95%CI: 1.84–5.62) phenotypes as compared with metabolically healthy normal weight (MHNW). In all, 41.3% of people with general obesity had 1 or 2 metabolic risk factors. Both groups had greater risk of ischemic stroke than MHNW people. Sensitivity analyses (considering abdominal obesity instead of general obesity) gave similar findings as the main analyses.

**Conclusion:**

Both MUO and MUNW phenotypes are risk factors of ischemic stroke. Early identification and targeted management of MUNW phenotype may have potential clinical implication for future stroke prevention and control.

## Introduction

Globally, stroke was the second-leading cause of death in 2023 (1). In 2019, China recorded 3.94 million new stroke cases, marking an 86.0% increase in the incidence rate since 1990 (2). With the aging of the population, the burden of stroke is expected to increase, and the morbidity associated with stroke will cause physical disability, with excessive medical resources used and increased healthcare costs.

Obesity, which is often accompanied by metabolic syndrome (MetS), is a modifiable risk factor of stroke (3, 4). A meta-analysis based on 1.8 million participants from 97 cohort studies found that 76% of the effect of body mass index (BMI) on risk of stroke was mediated by metabolic risk factors including blood pressure, glucose, and cholesterol levels (5). However, not all obese individuals exhibited MetS but had a healthy metabolic profile, called “metabolically healthy obesity” (MHO) (6). Correspondingly, a group of lean individuals tended to have a cluster of metabolic risk factors, called metabolically unhealthy normal weight (MUNW). Taking both MHO and MUNW phenotypes into consideration in clinical work and understanding their effect on stroke risk are necessary for stroke prevention and control.

Nevertheless, most previous studies focused on the association of MHO and cardiovascular disease (CVD), often considered coronary heart disease, heart failure, stroke, and CVD death together as the primary outcome, instead of examining stroke alone; thus, whether MHO is specifically associated with increased risk of stroke remains unclear (7). The Whitehall II cohort study reported that MHO individuals were at increased risk of stroke as compared with metabolically healthy normal weight (MHNW) individuals (8). However, data from US, Spanish and Korean populations showed conflicting results (9–11). Moreover, obesity usually begins with 1–2 MetS components, and non-obesity also seems to be accompanied by varying degrees of MetS, but few studies have investigated the influence of an accumulated number of metabolic risk factors on stroke risk across BMI status (12, 13).

Considering that the risks of ischemic stroke (accounts for 80% of stroke) among MHO and MUNO people were still not clear and studies focused on MHO–ischemic stroke association in Chinese population were limited (14, 15). Thus, we aimed to explore the association of MetS–BMI categories and the incidence of ischemic stroke in a rural Chinese population. As a secondary aim, we investigated the association of BMI status with different numbers of metabolic risk factors and risk of ischemic stroke.

## Materials and methods

### Study population

The Rural Chinese Cohort Study (RCCS) is a population-based prospective cohort study started during 2007 and 2008 with 20,194 participants randomly recruited from Xin'an county, Luoyang, Henan province, in the middle of China (16). The follow-up examination was conducted during 2013 and 2014. Detailed descriptions of the study design, study participants and data collection method were published previously (17). Briefly, participants aged ≥18 years who were free of severe psychological disorders, Alzheimer's disease, dementia, AIDs or other infectious diseases underwent questionnaire interview, anthropometric measurements, physical examination and laboratory measurements at both baseline and follow-up examinations. The study protocol was approved by the Medical Ethics Committee of Zhengzhou University, and all study participants gave their signed informed consent.

For the scope of the present study, we excluded participants who (1) had missing data on physical examination and anthropometric and laboratory measurements (*N* = 57) at baseline; (2) were underweight (BMI < 18.5 kg/m^2^; *N* = 709) at baseline; and (3) had history stroke and cardiovascular diseases (myocardial infarction and heart failure; *N* = 1,004) at baseline; (4) were lost to follow-up (*N* = 3,717). Finally, 14,707 eligible study participants were included to assess the association of metabolically healthy and obesity status and ischemic stroke risk.

### Data collection

At both baseline and follow-up examinations, study participants completed a designed questionnaire asking about socio-demographic characteristics (age, sex, marital status, monthly income, and education level), lifestyle (smoking, alcohol drinking, tea consumption, physical activity, and sleep duration), family and individual history of diseases and medication history by face-to-face interviews. Smoking was defined as smoking at least 100 cigarettes during the lifetime and was classified as current/ever smokers and never smokers. Alcohol drinking was defined as consuming alcohol 12 or more times in the last year and was classified as current/ever drinkers and never drinkers. Participants who drank tea more than 3 times a week for 6 months were defined as tea drinkers (18). Physical activity was classified as low, moderate or high according to the International Physical Activity Questionnaire (IPAQ) (19). Sleep duration was defined as the total sleep duration in a day including day and night.

Participants were told to wear light clothing during anthropometric measurements. Weight, height and waist circumference (WC) were measured twice by well-trained investigators. Weight was measured to the nearest 0.5 kg on a vertical weight scale. Height was measured to the nearest 0.1 cm with participants standing erect in bare feet. With participants gently breathing, WC was measured at the mid-point between the lowest rib and the iliac crest to the nearest 0.1 cm. BMI was calculated as weight (kg) divided by height (m) squared. Blood pressure and resting heart rate were measured 3 times, at an interval of 30 s, with an electronic sphygmomanometer (HEM-770AFuzzy, Omron, Japan), and the average of measurements was used for systolic blood pressure (SBP) and diastolic blood pressure (DBP). Overnight fasting blood samples were collected for assessing levels of fasting plasma glucose (FPG), total cholesterol (TC), triglycerides (TG) and high-density lipoprotein cholesterol (HDL-C). Low-density lipoprotein cholesterol (LDL-C) was calculated by the Friedewald formula (20).

### Definition of metabolically healthy and obesity status

Metabolically health status was based on MetS criteria (21). Considering that BMI was highly correlated with WC, most researchers suggest excluding WC among the MetS criteria when defining MHO according to the harmonized definition (21). Metabolically healthy was defined as having 0 of the 4 MetS criteria (blood pressure and FPG, TG and HDL-C). If participants had 1–4 of the MetS criteria, they were considered metabolically unhealthy. Obesity status was classified as normal weight (BMI 18.5–24 kg/m^2^), overweight (BMI 24–28 kg/m^2^) and general obesity (BMI ≥ 28 kg/m^2^) based on Chinese criteria (22). According to metabolically healthy and obesity status, 6 specific obesity phenotypes were further identified: metabolically healthy normal weight (MHNW), metabolically healthy overweight (MHOW), MHO, MUNW, metabolically unhealthy overweight (MUOW), and metabolically unhealthy obesity (MUO; Table 1).

**Table 1:** Harmonized definition of metabolically healthy and obesity status.

**Metabolically healthy status [based on MetS criteria (34)]**	**Metabolically healthy: Meeting 0 of MetS criteria indicated below (WC excluded): 1. SBP ≥ 130 mmHg and/or DBP ≥ 85 mmHg and/or use of anti-hypertensive medication and/or self-reported history of hypertension; 2. FPG ≥ 5.6 mmol/L and/or current use of anti-diabetic medication and/or self-reported history of diabetes; 3. TG ≥ 1.7 mmol/L or current use of lipid-lowering medication; 4. HDL-C < 1.0 mmol/L for men and < 1.3 mmol/L for women or current use of reduced HDL-C medication**
	Metabolically unhealthy: Meeting 1–4 of MetS criteria indicated above (WC excluded)
Obesity status [According to BMI based on Chinese criteria (22)]	1. **Normal weight**: BMI 18.5–24 kg/m^2^; 2. **Overweight**: BMI 24–28 kg/m^2^; 3. **General obesity**: BMI ≥ 28 kg/m^2^
**Metabolically health and obesity status**
1. Metabolically healthy normal weight (MHNW): normal weight with 0 MetS criteria (WC excluded)	
2. Metabolically healthy overweight (MHOW): overweight with 0 MetS criteria (WC excluded)	
3. Metabolically healthy obesity (MHO): general obesity with 0 MetS criteria (WC excluded)	
4. Metabolically unhealthy normal weight (MUNW): normal weight with 1–4 MetS criteria (WC excluded)	
5. Metabolically unhealthy overweight (MUOW): overweight with 1–4 MetS criteria (WC excluded)	
6. Metabolically unhealthy obesity (MUO): general obesity with 1–4 MetS criteria (WC excluded)	

MetS, metabolic syndrome; WC, waist circumference; SBP, systolic blood pressure; DBP, diastolic blood pressure; FPG, fasting plasma glucose; TG, triglycerides; HDL-C, high-density lipoprotein cholesterol.

### Assessment of ischemic stroke

Participants were followed up for fatal and non-fatal ischemic stroke event during the follow-up examination. If participants reported they had stroke, investigators further confirmed the type of stroke by checking the medical records and certificates of diagnosis (CT or MRI scans) (23), or verified it with relatives and village physicians. Participants who failed to provide reliable certificates of diagnosis were considered as uncertain type of stroke and were excluded in current analyses. Fatal ischemic stroke was collected by cause-of-death surveillance system.

### Statistical analysis

Baseline characteristics of the study participants are summarized as mean ± SD for continuous variables and number (percentage) for categorical variables. ANOVA test or chi-square test was used to test differences in baseline variables across 6 MetS–BMI categories. Cox regression analysis was used to calculate the hazard ratio (HR) and 95% confidence intervals (CIs) of incident ischemic stroke associated with different metabolically healthy and obesity status. We established 2 models to adjust for baseline confounding factors: (1) Model 1: age and sex; and (2) Model 2: age, sex, marital status, monthly income, education, smoking, alcohol drinking, tea consumption, physical activity, family history of stroke, resting heart rate, sleep duration, and LDL-C level. We further grouped participants according to the number of metabolic risk factors and obesity status, and considered MHNW as the reference.

To assess whether some traditional risk factors of stroke (age, sex, smoking, alcohol drinking, and physical activity) would influence the results, we further added the interaction terms of the 6 obesity phenotypes ^*^ risk factors to Model 2. To investigate the association of metabolically healthy and abdominal obesity status and incident ischemic stroke, we additionally used WC to define abdominal obesity according to Chinese criteria (24) instead of BMI and re-estimated the HRs for incident ischemic stroke associated with MetS–WC categories as a sensitivity analysis. The definition of MHO based on WC were presented in Supplementary Table 1.

All statistical analyses were performed with SAS v9.4 and all reported *p* values were 2-sided, with *p* < 0.05 considered statistically significant.

## Results

### Baseline characteristics

The prevalence of overweight and general obesity in metabolically healthy individuals was 21.9% and 3.7%, and in the metabolically unhealthy group was 39.3% and 17.4%. Among different MetS–BMI categories, significant differences were found in terms of age, sex, marital status, education level, smoking, alcohol drinking, tea consumption, physical activity, family history of stroke, and sleep duration (all *p* < 0.05; Table 2). Anthropometric and laboratory measurements including BMI, WC, resting heart rate, SBP, DBP, FPG, TC, TG, HDL-C, and LDL-C also showed significant differences across groups (all *p* < 0.05).

**Table 2:** Baseline characteristics of 14,631 eligible study participants by metabolically healthy and BMI groups.

**Baseline characteristics**	**Metabolically healthy**			**Metabolically unhealthy**			***p***
	**Normal weight (*****N*** = **1,702)**	**Overweight (*****N*** = **501)**	**General obesity (*****N*** = **84)**	**Normal weight (*****N*** = **5,383)**	**Overweight (*****N*** = **4,875)**	**General obesity (*****N*** = **2,162)**	
Age, years	45.4 (13.3)	46.4 (10.5)	45.7 (9.8)	50.7 (13.8)	50.9 (11.7)	50.5 (11.1)	< 0.001
Men	962 (56.5)	222 (44.3)	27 (32.1)	2,176 (40.4)	1,645 (33.7)	561 (25.9)	< 0.001
Married	1,534 (90.1)	478 (95.4)	78 (92.9)	4,788 (88.9)	4,510 (92.5)	2,018 (93.3)	< 0.001
High school or above	237 (13.9)	58 (11.6)	8 (9.5)	585 (10.9)	487 (10.0)	196 (9.1)	< 0.001
Monthly income >500 Yuan	140 (8.2)	31 (6.2)	4 (4.8)	336 (6.2)	328 (6.7)	158 (7.3)	0.179
Smoking	709 (41.7)	165 (32.9)	17 (20.2)	1,559 (29.0)	1,146 (23.5)	394 (18.2)	< 0.001
Alcohol drinking	315 (18.5)	89 (17.8)	9 (10.7)	554 (10.3)	549 (11.3)	226 (10.5)	< 0.001
Tea consumption	369 (21.7)	99 (19.8)	25 (29.8)	952 (17.7)	948 (19.4)	398 (18.4)	0.001
Physical activity							< 0.001
Low	396 (23.3)	116 (23.2)	24 (28.6)	1,614 (30.0)	1,479 (30.3)	763 (35.3)	
Moderate	339 (19.9)	82 (16.4)	17 (20.2)	1,131 (21.0)	1,135 (23.3)	527 (24.4)	
High	967 (56.8)	303 (60.5)	43 (51.2)	2,638 (49.0)	2,261 (46.4)	872 (40.3)	
Family history of stroke	188 (11.0)	70 (14.0)	6 (7.1)	687 (12.8)	783 (16.1)	299 (13.8)	< 0.001
Sleep duration, h	8.5 (1.5)	8.4 (1.3)	8.2 (1.4)	8.6 (1.6)	8.7 (1.6)	8.7 (1.6)	< 0.001
BMI, kg/m^2^	21.3 (1.5)	25.5 (1.1)	29.7 (1.8)	21.8 (1.4)	25.8 (1.1)	30.3 (2.1)	< 0.001
WC, cm	74.1 (5.7)	84.8 (6.1)	93.6 (7.2)	76.3 (6.1)	86.8 (6.2)	96.9 (7.3)	< 0.001
RHR, beats/min	72.9 (10.6)	72.2 (9.4)	73.6 (7.9)	75.1 (11.1)	75.1 (10.4)	75.9 (9.8)	< 0.001
SBP, mmHg	111.8 (9.5)	114.2 (8.7)	116.4 (7.8)	124.0 (20.0)	129.1 (19.9)	134.5 (21.1)	< 0.001
DBP, mmHg	70.1 (6.8)	73.1 (6.2)	75.5 (5.2)	76.6 (10.9)	81.1 (10.7)	85.7 (11.4)	< 0.001
FPG, mmol/L	5.0 (0.4)	5.0 (0.4)	5.1 (0.4)	5.6 (1.4)	5.8 (1.6)	6.0 (1.7)	< 0.001
TC, mmol/L	4.2 (0.7)	4.4 (0.8)	4.5 (0.7)	4.3 (0.9)	4.6 (1.0)	4.7 (0.9)	< 0.001
TG, mmol/L	1.0 (0.3)	1.0 (0.3)	1.1 (0.3)	1.5 (0.9)	2.0 (1.3)	2.2 (1.4)	< 0.001
HDL-C, mmol/L	1.4 (0.2)	1.4 (0.2)	1.4 (0.2)	1.1 (0.2)	1.1 (0.2)	1.1 (0.2)	< 0.001
LDL-C, mmol/L	2.4 (0.7)	2.6 (0.7)	2.7 (0.7)	2.5 (0.7)	2.7 (0.8)	2.7 (0.8)	< 0.001

Continuous data are shown as mean (SD) and categorical data as number (percentage). WC, waist circumference; RHR, resting heart rate; SBP, systolic blood pressure; DBP, diastolic blood pressure; FPG, fasting plasma glucose; TC, total cholesterol; TG, triglycerides; HDL-C, high-density lipoprotein cholesterol; LDL-C, low-density lipoprotein cholesterol.

### Obesity phenotype and incident ischemic stroke

During a median follow-up of 6.02 years, 522 newly diagnosed ischemic stroke cases occurred. Among metabolically healthy participants, the incidence of ischemic stroke was 1.12%, 1.40%, and 1.19% for MHNW, MHOW, and MHO groups, respectively (Table 3). In the metabolically unhealthy group, the incidence was 3.60%, 4.21%, and 4.44% for MUNW, MUOW, and MUO groups, respectively. Adjusted risk of ischemic stroke was increased with MUNW (HR: 2.36, 95%CI: 1.40–3.98), MUOW (HR: 3.23; 95%CI: 1.92–5.43), and MUO (HR: 3.22, 95%CI: 1.84–5.62) as compared with MHNW. No significant association was found between MHO and ischemic stroke (HR: 1.87; 95%CI: 0.25–14.18). On interaction analysis, the association of obesity phenotypes and ischemic stroke was not further modified by sex, age, smoking, alcohol drinking, and physical activity (all *p*_interaction_ > 0.05, data not shown).

**Table 3:** Association of metabolically healthy and unhealthy BMI groups with incident ischemic stroke.

**Metabolically health status**	**BMI status**	**No. of cases**	**Incidence (%)**	**RR^a^ (95%CI)**	**RR^b^ (95%CI)**
Metabolically healthy	Normal weight	19	1.12	1.00	1.00
	Overweight	7	1.40	1.42 (0.60–3.39)	0.92 (0.31–2.77)
	General obesity	1	1.19	1.36 (0.18–10.20)	1.87 (0.25–14.18)
Metabolically unhealthy	Normal weight	194	3.60	2.49 (1.55–4.01)	2.36 (1.40–3.98)
	Overweight	205	4.21	3.28 (2.04–5.27)	3.23 (1.92–5.43)
	General obesity	96	4.44	3.73 (2.27–6.14)	3.22 (1.84–5.62)

RR, relative risk; CI, confidence interval. ^a^adjusted for age and sex. ^b^adjusted for age, sex, marital status, monthly income, education, smoking, alcohol drinking, tea consumption, physical activity, family history of stroke, resting heart rate, sleep duration, and low-density lipoprotein cholesterol.

**Figure 1:**
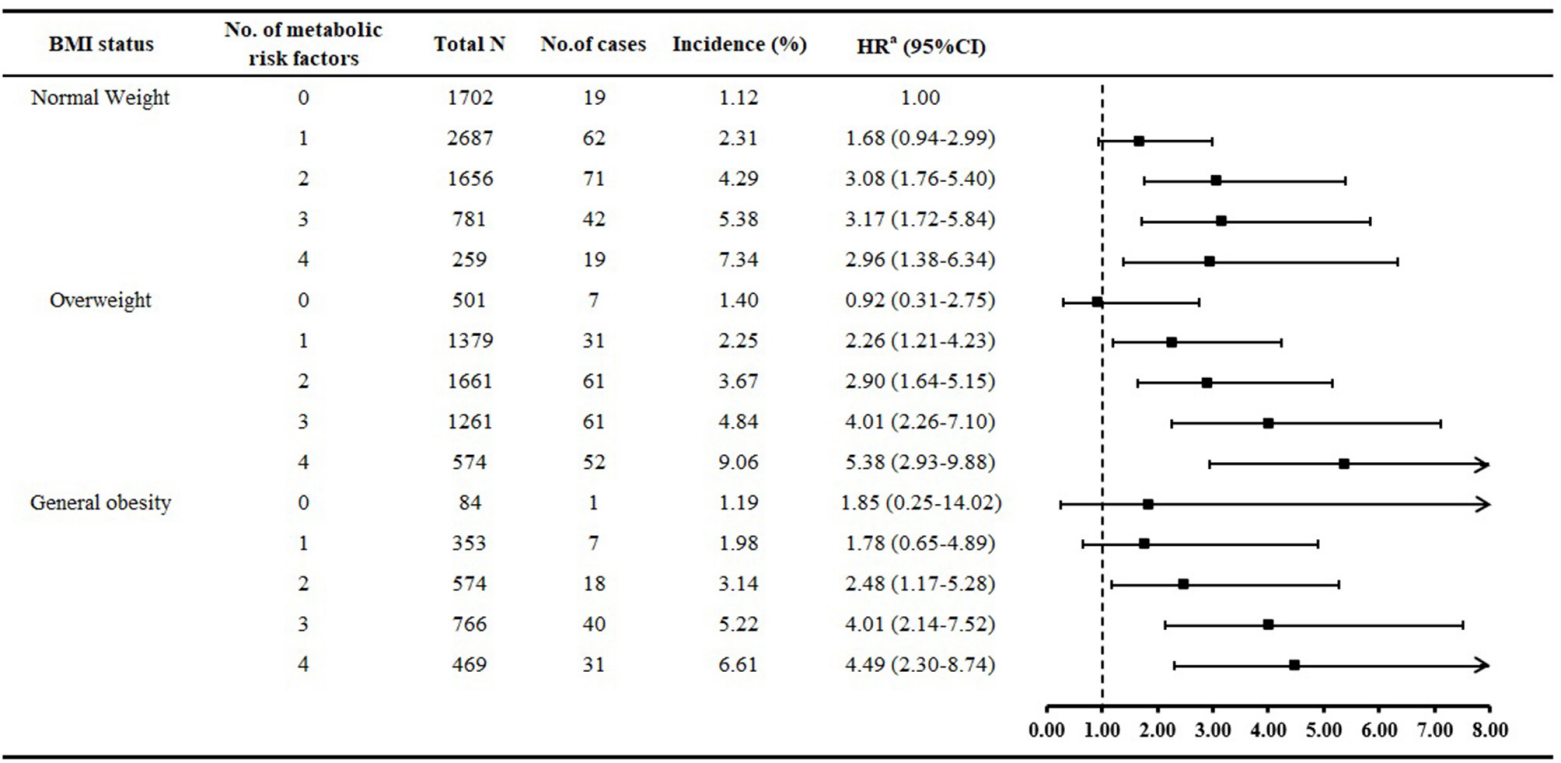
Multivariate relative risk of ischemic stroke associated with number of metabolic risk factors in different BMI groups. ^a^Adjusted for age, sex, marital status, monthly income, education, smoking, alcohol drinking, tea consumption, physical activity, family history of stroke, resting heart rate, sleep duration, and low-density lipoprotein cholesterol.

Figure 1 shows that the incidence of ischemic stroke increased with increased number of metabolic risk factors. Overall, 41.3% of people with general obesity had 1 or 2 metabolic risk factors, and both groups showed greater risk of ischemic stroke than MHNW people. For overweight individuals, those with 1 or 2 metabolic risk factors, risk of ischemic stroke was 2.26- and 2.90-fold increased as compared with MHNW individuals. Risk of ischemic stroke was increased for normal-weight people with 2–4 metabolic risk factors than those with metabolically healthy status.

### Sensitivity analysis

**Figure 2:**
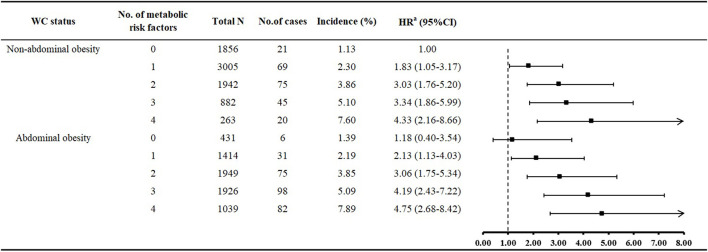
Multivariate relative risk of ischemic stroke associated with number of metabolic risk factors in different waist circumference (WC) groups. ^a^Adjusted for age, sex, marital status, monthly income, education, smoking, alcohol drinking, tea consumption, physical activity, family history of stroke, resting heart rate, sleep duration, and low-density lipoprotein cholesterol.

When we used WC to define abdominal obesity, we found similar results as for the main analysis (Table 4). 6.4% abdominal obese people was in metabolically healthy condition. Adjusted risk of ischemic stroke was increased with metabolically healthy abdominal obesity vs. metabolically healthy non-abdominal obesity, however, this association was not statistically significant (HR: 1.17; 95%CI: 0.39–3.51). The risk of ischemic stroke was 2.54-fold increased with metabolically unhealthy but non-abdominal obesity (HR: 2.54, 95%CI: 1.54–4.20); and the highest risk was with metabolically unhealthy abdominal obesity (HR: 3.37, 95%CI: 2.02–5.61). Risk of ischemic stroke was significantly increased with non-abdominal obesity with accumulated number of MetS risk factors as compared with no metabolic risk factors (Figure 2). Except for no MetS risk factor, the risk of ischemic stroke was increased with abdominal obesity regardless of MetS risk-factor status.

**Table 4:** Association of different metabolically healthy and obesity status groups (defined by waist circumference) with incident ischemic stroke.

**Metabolic obesity phenotypes**	**Total N**	**No. of cases**	**Incidence (%)**	**RR^a^ (95%CI)**	**RR^b^ (95%CI)**
Metabolically healthy non-abdominal obesity	1,856	21	1.13	1.00	1.00
Metabolically healthy abdominal obesity	431	6	1.39	1.42 (0.57–3.52)	1.17 (0.39–3.51)
Metabolically unhealthy non-abdominal obesity	6,092	209	3.43	2.47 (1.58–3.88)	2.54 (1.54–4.20)
Metabolically unhealthy abdominal obesity	6328	286	4.52	3.52 (2.23–5.53)	3.37 (2.02–5.61)

RR, relative risk; CI, confidence interval. ^a^adjusted for age and sex. ^b^adjusted for age, sex, marital status, monthly income, education, smoking, alcohol drinking, tea consumption, physical activity, family history of stroke, resting heart rate, sleep duration, and low-density lipoprotein cholesterol.

## Discussion

In the present study, 15.3% of rural Chinese people had general obesity and 3.7% of these were “metabolically healthy”. We also observed a large proportion of the MUNW phenotype, which was a major risk factor for ischemic stroke, among normal-weight individuals (76.0%). Furthermore, although the lowest and highest risks of ischemic stroke were not always in people with 0 and 4 metabolic risk factors, respectively, our results suggest to some extent that excess metabolic risk factors is associated with increased risk of ischemic stroke regardless of obesity status. These findings were replicated when using WC to define abdominal obesity in a sensitivity analysis. Our findings indicated that weight loss programs should be emphasized for obese people regardless of their metabolically healthy status. Behavior modification and medical therapy aiming to control blood pressure, glucose and lipid profiles may have potential and efficient benefit for stroke prevention in normal-weight people.

Early and precise identification and modification of risk factors is imperative for stroke prevention (25, 26). Although epidemiological studies have identified a series of modifiable risk factors (smoking, obesity, hypertension, diabetes, and dyslipidemia) for stroke and targeted intervention programs have been implemented, the global burden of stroke is still high, with a large impact in developing countries (27). The subset of MHO phenotype has been identified, but the association of MHO and ischemic stroke was still not well documented and remained controversial (28). A recent meta-analysis based on 43 cohort studies reported a significant linear dose–response relation between BMI and CVD risk among metabolically healthy individuals (7). However, most studies considered stroke as one component of CVD events and did not further consider the association of MHO and different types of stroke (29–31). Results from the Atherosclerosis Risk in Communities study of 14,658 US people demonstrated that during a mean follow-up of 18.7 years, MHO individuals had the lowest risk of stroke (9). Similar results were found in Spanish and Korean studies (10, 11). In contrast, the Whitehall II cohort study first reported a positive association between MHO and stroke among 7,122 civil servants aged 39–63 years during a median follow-up of 17.4 years, but the findings may not generalize to a general population (8). Another large-scale cohort study of 3.5 million UK people with 5.4 years follow-up also showed MHO associated with a 1.07-fold risk of stroke as compared with MHNW (13). However, both studies did not distinguish the type of stroke.

A cross-sectional study aiming to explore the MHO–stroke association in a Chinese population reported that the prevalence of ischemic stroke was lowest with MHO (1.7%) but higher with MUNO (4.9%) and MUO (4.8%), and MHO was not significantly associated with ischemic stroke (32). Results from the Kailuan cohort reported a positive association between MUNW and stroke among occupational population; however, the association between MHO and stroke was not statistically significant (33). Similar results were also found in another Chinese cohort study, which was based on 34,294 community residents (15). Both aforementioned Chinese cohort studies did not distinguish the risk of ischemic stroke. Our study first generated this research question in a Chinese population by using a prospective cohort study design and reported both MUO and MUNW associated with increased risk of ischemic stroke. We also observed a positive but not significant association between MHO and ischemic stroke. The reason may due to the short-term follow-up or small proportion of the MHO phenotype. Further studies based on various populations are still needed to replicate our findings.

Additionally, most studies used BMI to define MHO because BMI is highly correlated with WC (21, 34). However, previous studies demonstrated that Asian populations tend to have lower BMI but higher WC as compared with Western populations because the body fat is commonly distributed in the abdominal cavity (35). Thus, metabolically healthy abdominal obesity may be more prevalent in Chinese populations. Therefore, we conducted a sensitivity analysis and found similar results as main analyses. Our findings implied that obesity or metabolically unhealthy status were associated with future development of ischemic stroke. MHO may not be a benign condition and should not be considered “healthy”. Management of weight and WC is recommended for MHO individuals in terms of stroke prevention.

Of note, the inconsistent aforementioned results may be related to several factors, such as the definition of MHO, different populations and follow-up times or other confounding factors. Particularly, the definition of MHO may be the major issue that leads to the controversial findings (36). Some investigators suggest that MHO should refer to obese people without any metabolic risk factors (21, 37); however, most studies commonly used the less strict MHO definitions: obese people with 1 or 2 MetS risk factors (7). Our study, using a strict criteria definition, found only 3.7% of obese individuals considered “metabolically healthy”. If using the Adult Treatment Panel-III (ATP-III) definition (38), with < 2 MetS risk factors considered metabolically healthy, more general obese participants would exhibit the MHO phenotype. In contrast, nearly three quarters of normal-weight people had varying degrees of metabolic abnormality. Thus, the impact of the number of metabolic risk factors on stroke risk should be clarified across different BMI status groups. A UK study reported increased risk of stroke with increased number of metabolic abnormalities in normal-weight, overweight, and general obese groups, which was in line with our study (13). In the present study, the incidence rate basically increased with increasing number of MetS risk factors and the HR increased accordingly regardless of obesity status defined by BMI or WC. Future studies using a strict definition of MHO are still needed to compare with our results.

Our study was based on a prospective cohort design with a large sample size and well-controlled covariates, which could provide high-quality and reliable evidence. Furthermore, although numerous studies emphasized the weakness of the MHO definitions [i.e. ATP III (38), Karelis and Rabasa-Lhoret (39), and Wildman et al. (40)] and recommended considering metabolically healthy strictly with 0 of the 4 MetS criteria (WC excluded), most previous studies still used a less reasonable definition. Our study chose a highly recognized definition of MHO and also estimated the influence of MetS risk factors and BMI combination on risk of ischemic stroke, which could provide more detailed evidence for stroke prevention.

However, our study has several limitations. First, the study was based on a rural Chinese population from one county in the middle of China, so the current results may not be generalized to other populations. Future studies are still needed to estimate the MHO–stroke association in multi-ethnic and multicenter populations. Second, MHO seems to be a transient status of MUO. Because we completed only one follow-up examination, we could not investigate whether dynamic metabolically healthy and obesity status were associated with risk of ischemic stroke. Third, although we built models to adjust confounding factors associated with stroke, there is still a possibility of residual confounding caused by other covariates, such as psychological factors, medication for diseases, and other unknown factors.

## Conclusion

In conclusion, our data showed increased risk of incident ischemic stroke for MUNW and MUO individuals. An excess number of metabolic risk factors was positively associated with risk of ischemic stroke. The term MHO may not be suitable because of its related unhealthy outcome. The identification and management of MUNW phenotype may have potential clinical implications for future stroke prevention and control.

## Data Availability

The raw data supporting the conclusions of this article will be made available by the corresponding author, without undue reservation.
