# Supplementary material for: Metabolic obesity phenotypes and risk of ischemic stroke: The Rural Chinese Cohort Study
Source: Front Nutr. 2026 Jan 12;12:1749472. doi: 10.3389/fnut.2025.1749472 (PMC12833963; doi:10.3389/fnut.2025.1749472)
Supplement: Supplementary file 1 [file Table_1.doc]

| **Supplementary Table 1. Harmonized definition of metabolically healthy and obesity status** | |
| --- | --- |
| **Metabolically healthy status**  (based on MetS criteria^[1]^) | **Metabolically healthy**:  Meeting 0 of MetS criteria indicated below (WC excluded): |
|  | 1. SBP ≥130 mmHg and/or DBP ≥85 mmHg and/or use of anti-hypertensive medication and/or self-reported history of hypertension;  2. FPG ≥5.6 mmol/L and/or current use of anti-diabetic medication and/or self-reported history of diabetes;  3. TG ≥1.7 mmol/L or current use of lipid-lowering medication;  4. HDL-C <1.0 mmol/L for men and <1.3 mmol/L for women or current use of reduced HDL-C medication. |
|  | **Metabolically unhealthy**:  Meeting 1-4 of MetS criteria indicated above (WC excluded) |
| **Obesity status**  **(**According to WC based on Chinese criteria^[2]^**)** | 1. **Non-abdominal obesity**: men: WC<90cm, women: WC<80cm  2. **Abdominal obesity**: men≥90cm, women≥80cm |
| **Metabolically health and obesity status** | |
| 1. Metabolically healthy non-abdominal obesity: non-abdominal with 0 MetS criteria  2. Metabolically healthy abdominal obesity: abdominal obesity with 0 MetS criteria  3. Metabolically unhealthy non-abdominal obesity: non-abdominal obesity with 1-4 MetS criteria  4. Metabolically unhealthy abdominal obesity: abdominal obesity with 1-4 MetS criteria | |
| MetS: metabolic syndrome; WC: waist circumference; SBP: systolic blood pressure; DBP: diastolic blood pressure; FPG: fasting plasma glucose; TG: triglycerides; HDL-C: high-density lipoprotein cholesterol. | |

**References**

1. Alberti KG, Eckel RH, Grundy SM, Zimmet PZ, Cleeman JI, Donato KA, et al. Harmonizing the metabolic syndrome: a joint interim statement of the International Diabetes Federation Task Force on Epidemiology and Prevention; National Heart, Lung, and Blood Institute; American Heart Association; World Heart Federation; International Atherosclerosis Society; and International Association for the Study of Obesity. Circulation. 2009;120(16):1640-1645.
2. Zhou BF, Cooperative Meta-Analysis Group of the Working Group on Obesity in C. Predictive values of body mass index and waist circumference for risk factors of certain related diseases in Chinese adults--study on optimal cut-off points of body mass index and waist circumference in Chinese adults. Biomed Environ Sci. 2002;15(1):83-96.
